# 1490. Predictors of Early Mortality in HIV-associated Tuberculous Meningitis

**DOI:** 10.1093/ofid/ofad500.1325

**Published:** 2023-11-27

**Authors:** Enock Kagimu, Ananta Bangdiwala, John Kasibante, Mable Kabahubya, Jane Gakuru, Mugabi Timothy, Suzan Namombwe, Sarah Kimuda, Derrick Kasozi, Morris K Rutakingirwa, Kenneth Ssebambulidde, Lillian Tugume, Edwin Nuwagira, Samuel Okurut, Laura Nsangi, Mucunguzi Atukunda, Jayne P Ellis, Darlisha A Williams, Abdu Kisekka Musubire, David R Boulware, David Meya, Nathan C Bahr, Fiona V Cresswell

**Affiliations:** Infectious Diseases Institute, Makerere University, Kampala, Kampala, Uganda; University of Minnesota, Minneapolis, Minnesota; Infectious Diseases Institute, Makerere University, Kampala, Kampala, Uganda; Infectious diseases Institute, Makerere University, College of Health Sciences, Kampala, Kampala, Uganda; Infectious Diseases Institute, Makerere University, Kampala, Kampala, Uganda; Infectious diseases institute, Kampala, Kampala, Uganda; Infectious Diseases Institute, Kampala, Kampala, Uganda; Infectious diseases Institute, Makerere University, College of Health Sciences, Kampala, Kampala, Uganda; Infectious Diseases Institute, Kampala, Kampala, Uganda; Makerere University, College of Health sciences, Kampala, Kampala, Uganda; Infectious Diseases Institute, Makerere University, Kampala, Kampala, Uganda; Infectious Diseases Institute, Makerere University, Kampala, Kampala, Uganda; Mbarara University of Science and Technology, Mbarara city, Mbarara, Uganda; Infectious Diseases Institute, Makerere University, Kampala, Kampala, Uganda; Infectious Diseases Institute, Makerere University, Kampala, Kampala, Uganda; Infectious Diseases Institute, Makerere University, Kampala, Kampala, Uganda; london school of hygiene and tropical medicine, London, England, United Kingdom; University of Minnesota, Minneapolis, Minnesota; Infectious Diseases Institute, Makerere University, Kampala, Kampala, Uganda; University of Minnesota, Minneapolis, Minnesota; Infectious Diseases Institute, Makerere University, Kampala, Kampala, Uganda; University of Kansas, Kansas City, Kansas; London school of Hygiene and Tropical Medicine, London, England, United Kingdom

## Abstract

**Background:**

Tuberculous meningitis (TBM) mortality averages at 27% but may reach 70% in HIV co-infection. High MRC severity grade, lower cerebrospinal fluid (CSF) white blood cell count (WBC) count, low weight, low CD4 and low plasma sodium were linked to mortality in Vietnam. Prognostic factors in African adults with HIV-associated TBM are unknown. We sought to determine the baseline factors predictive of death in HIV-positive Ugandan adults with TBM.

**Methods:**

We prospectively enrolled patients who received TBM treatment and were classified as definite, probable or possible TBM by the uniform case definition from January 2019 to March 2023 in two National Referral Hospitals in Uganda. Participants received standard quadruple TB and corticosteroid therapy. We assessed association between baseline clinical and CSF characteristics (WBC< 5, 5-100, >100 cells/µl), antiretroviral therapy (ART) status and 14-day mortality.

**Results:**

In 261 participants, median age was 36 years, 46% were women, median CD4 count was 74 cells/µl. 27% had definite, 39% probable, and 34% possible TBM. 142 (54%) had baseline CSF WBC < 5, 50 (19%) had 5-100, and 69 (27%) had >100 cells/µl.

Overall, 14-day mortality was 25.9%. Mortality was 73.6% in participants with CSF WBC count < 5, 7.5% in 5-100, and 18.9% in >100 cells/µl, p=0.0012. Factors associated with 14-day mortality were MRC severity grade, CSF WBC, CSF opening pressure (OP), CSF protein and glucose, CD4 count. ART status did not influence survival. The hazard of 14-day mortality was 4 times as high for those with CSF WBC < 5 cells/µl as for those with >100 cells/ µl (95%CI 1.47-11.5, P=0.004). However, on adjustment for Glasgow Coma Scale (GCS), CSF OP, glucose, and CD4 count the association was less significant (aHR, 2.93, 0.82-10.4, P=0.13).

**Figure:**
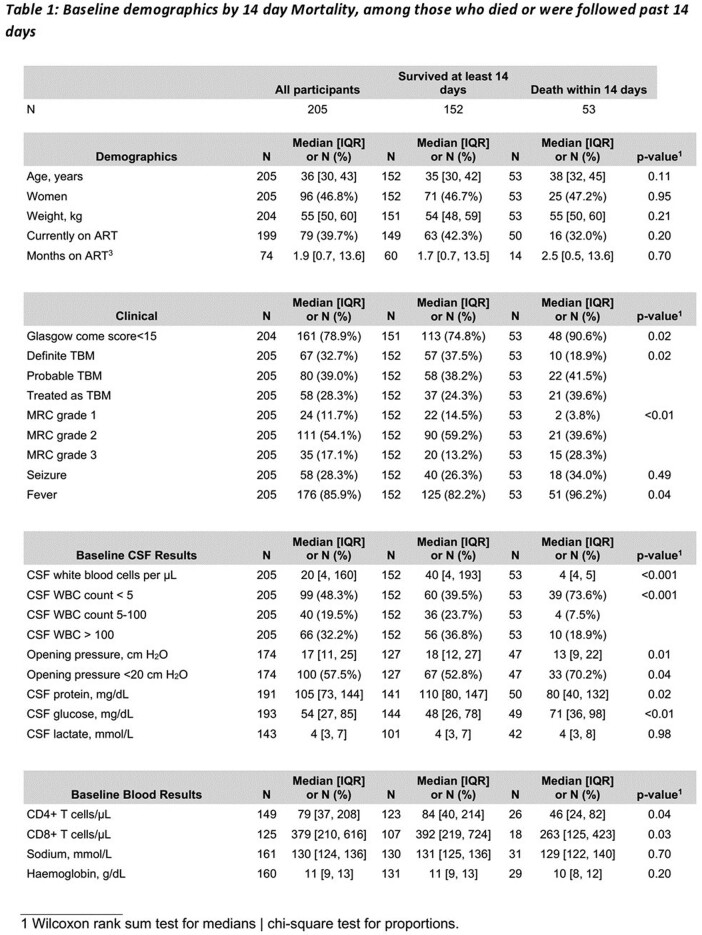

**Figure:**
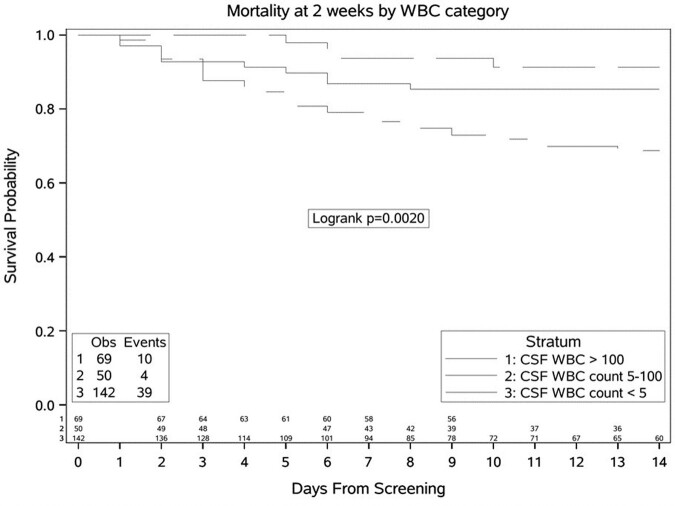

**Figure:**
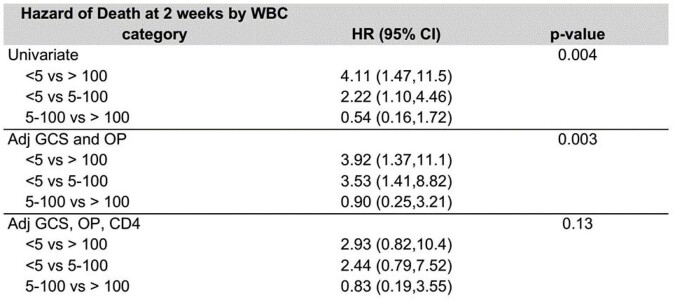

**Conclusion:**

14-day mortality varied significantly by baseline CSF WBC group, being extremely high (73.6%) in those with a paucity of CSF inflammation (WBC < 5 cells/µl). Those with intermediate inflammation (CSF WBC 5-100 cells/µl) had lowest mortality in-line with the damage response framework parabola. The association with acellular CSF and mortality was ameliorated by adjustment for CD4 count. CSF inflammation is at predictor of mortality in HIV-associated TBM and CSF WBC-directed corticosteroid therapy needs further exploration.

**Disclosures:**

**All Authors**: No reported disclosures

